# Reparative Mineralized Tissue Characterization after Direct Pulp Capping with Calcium-Silicate-Based Cements

**DOI:** 10.3390/ma12132102

**Published:** 2019-06-29

**Authors:** Xuan Vinh Tran, Hamideh Salehi, Minh Tam Truong, Minic Sandra, Jeremy Sadoine, Bruno Jacquot, Frédéric Cuisinier, Catherine Chaussain, Tchilalo Boukpessi

**Affiliations:** 1 EA 2496, Laboratory of Orofacial Pathologies, Imaging and Biotherapies, Dental School, Paris Descartes University, Sorbonne Paris Cite, 1 rue Maurice Arnoux, 92120 Montrouge, France; 2 Faculty of Odonto-Stomatology, University of Medicine and Pharmacy at Ho Chi Minh City (UMP), 217 Hong Bang street, Ward 11, Dist 5, Ho Chi Minh City, Viet Nam; 3 Bioengineering and Nanosience Laboratory EA4203, Montpellier University, 545 avenue du Presesseur Jean-Louis Viala, 34193 Montpellier CEDEX 5, France; 4 Thanh Vu Medic Bac Lieu Hospital; Highway 1 bypass, Ward 7, Bac Lieu City, Bac Lieu province, Viet Nam; 5 AP-HP Department of odontology, Charles Foix and Bretonneau Hospitals, 12 avenue de la République, 94200, Ivry-sur-Seine, France and 12 rue Carpeaux, 75018 Paris, France

**Keywords:** calcium silicate cement, Biodentine™, MTA, pulp capping, reparative dentinogenesis, Raman, SEM

## Abstract

Nowadays, the preservation of dental pulp vitality is an integral part of our daily therapies. The success of these treatments depends on the clinical situation as well as the biomaterials used. Mineral Trioxide aggregate and Biodentine^TM^ are commonly used as pulp capping materials. One objective of vital pulp therapy is the repair/regeneration of the pulp. In addition to the initial inflammatory status of the pulp, the nature and quality of the new mineralized tissue obtained after pulp capping directly influence the success of the treatment. In order to characterize the reparative dentin, in the current study, the chemical composition and microstructure of the dentin bridge after direct pulp capping using Biodentine™ and mineral trioxide aggregate (MTA) was studied by using Raman microspectroscopy and scanning electron microscopy, respectively. The results showed that the reparative dentin bridge observed in both groups presented dentin tubules and chemical composition similar to primary dentin. With the limitations of this study, the calcium-silicate-based cements used as pulp capping materials provide an optimal environment for pulp healing, resulting in a reparative dentin resembling on certain points of the primary dentin and the regeneration of the pulp.

## 1. Introduction

Dental pulp is a loose connective tissue enclosed within rigid dentin walls. Primary dentin is the tubular dentin formed by actively secreting primary odontoblasts during crown formation. Secondary dentin refers to the physiological dentin that is continuously deposited after the completion of tooth eruption. Tertiary dentin is formed in case of injury. Mild stimuli lead to a reactionary dentin synthetized by the existing odontoblasts. Stronger stimuli lead to the death of odontoblasts. Under favorable conditions, it is believed that dental pulp stem cells or other cells differentiate into odontoblast-like cells to produce reparative dentin. This reparative dentin is desired during pulp capping procedures. Nowadays, the preservation of pulp vitality is an integral part of our daily therapies. The success of these treatments depends on the clinical situation as well as the biomaterials used. Calcium hydroxide has been the only material used for vital pulp therapy for over a century. In the last two decades, hydraulic cements composed primarily of tricalcium silicate have been proposed for many clinical applications in dentistry, including direct pulp capping [1]. Mineral trioxide aggregate (MTA) composed of Portland and bismuth oxide is the most famous of these cements and has been shown to induce reparative dentinogenesis in mechanically exposed pulps [2,3]. However, it presents different drawbacks consisting in handling difficulty, extended setting time [4], capacity to induce crown discoloration, and poor mechanical properties. Several new calcium silicate-based materials have been developed [5,6], aiming to improve the disadvantages of MTA. Biodentine^TM^ (Septodont, Saint Maur des Fosses, France) is amongst these materials and is claimed to be used as a dentin replacement material, in addition to having endodontic indications similar to those of MTA. In fact, it is resin-free and mainly composed of pure tricalcium silicate. The chemical composition differs from MTA by the addition of calcium carbonate to the powder. The liquid is constituted of a hydrated calcium chloride (as an accelerator to reduce setting time) and a water reducing agent [7]. It has a shorter setting time of 12 min (according to the manufacturer’s data sheets), compared with the 2 h 45 min of MTA [8]. Biodentine^TM^ has shown better compression and surface properties than other tricalcium silicate-based materials [9]. The usefulness of this material as a restorative material as well as a direct pulp capping agent has been demonstrated in humans and in rats [3,10,11,12]. These two materials (MTA and Biodentine™) are well known and are both capable of inducing the formation of new mineralized tissue in continuity with secondary dentin [2]. What is really important to know is the real quality of this “reparative dentin”. In addition to the initial inflammatory status of the pulp, the nature and quality of the new mineralized tissue directly influence the success of the treatment. In fact, it has been reported that pulp response after direct capping is necessarily linked to bacterial microleakage [11,13]. Poor quality reparative dentin would allow bacterial diffusion between the material and the pulp tissue. It has been shown that pulp capping using calcium hydroxide causes tunnel defects inside the dentin bridge [3]. In the case of MTA and Biodentine^TM^, although the materials are often used successfully in the clinical setting, little is known about the characteristics of the new mineralized tissue. The characterization of this newly formed tissue is decisive to determine its tightness. Thus, in the current study, the chemical composition and microstructure of the dentin bridge after direct pulp capping using Biodentine™ and MTA were studied by using Raman microspectroscopy and scanning electron microscopy, respectively. A comparison was made with the primary dentin serving as the control dentin.

## 2. Materials and Methods

This study was designed according to the ARRIVE guidelines and was approved by the Animal Care Committee of Université Paris Descartes (protocol CEEA34.CC.016.11).

Fourteen male rats (6 weeks old) were purchased from OFA (Charles River Laboratories, Lille, France). Each animal received two compared treatments on the left (Biodentine™) and right (MTA) first maxillary molars. 

The animals were anesthetized by intraperitoneal injection of a combination of ketamine 10% (Imalgene 500, Merial, Lyon, France) and xylazine 2% (Rompun, Bayer, Puteaux, France). Aided by an operative microscope (Carl Zeiss, Oberkochen, Germany), 28 cavities were prepared on the mesial surfaces of the left and right maxillary first molars by means of a 0.6 mm diameter round carbide bur (Dentsply-Maillefer, Baillaigues, Switzerland). Pulps were exposed with a Protaper File F1 (Dentsply-Maillefer) and capped with either Biodentine (Lot no. B01095, Septodont, St Maur des Fosses, France) or MTA (Lot no. 08003394, white ProRoot MTA, Dentsply Tulsa Dental, Tulsa, OK, USA), mixed according to the manufacturer’s instructions. All cavities were subsequently restored with a light-cured composite (FloRestore; Denmat, Lompoc, CA 93436, USA). Animals were then allowed to recover. After 30 days, animals were anesthetized and sacrificed by an intracardiac perfusion with 4% paraformaldehyde buffered with sodium cacodylate 0.1 M at pH 7.2–7.4.

### 2.1. Sample Preparation

Block sections including molars were dissected from the maxilla and immersed in the fixative solution (4% paraformaldehyde) for 24 h at 4 °C. Then, the samples were prepared for histology or SEM and Raman spectroscopy. 

The pulp healing process induced by either Biodentine^TM^ or MTA was evaluated by using hematoxylin eosin (HE) staining. For this purpose, tissue demineralization was performed at 4 °C with constant agitation in 4.13% EDTA (pH 7.4) by changing the solution three times a week for approximately 4 months. After tissue was embedded in Paraplast, serial sections (7 µm) were cut with a microtome (Jung AG, Heidelberg, Germany) in a mesiodistal direction. Sections were stained for HE. 

For SEM and Raman analysis, only Biodentine™ and MTA groups were used. Tissue was embedded in methacrylate resin. Resin blocks were cut using a diamond blade (Accutom-5, Struers) in a mesiodistal direction and polished with silicon carbide papers with increasing grain (800, 1200, 2400, and 4000 grit) under copious irrigation. The surfaces were then polished with soft tissue discs with fine diamond suspensions in combination with DL-lubricant cooling solution. 

### 2.2. SEM and EDX Analysis

The pieces were incubated in a solution of 5% sodium hypochlorite for 5 min and sonicated again. They were dried with alcohol and then with a vacuum desiccator. The specimens were either sputter-coated with gold palladium for SEM morphological observation (Cambridge S260, Cambridge, UK) or coated with carbon deposit for EDX (Bruker AXS, Karlsruhe, Germany). For EDX, the signals in the energy region for phosphorus (P) and calcium (Ca) were recorded and compared between the secondary dentin and the reparative dentin bridge on the same sample. The samples were analyzed for every group and three analysis points were obtained for every area of interest (secondary dentin or reparative dentin bridge). The results were the averages of the data obtained.

### 2.3. Confocal Raman Microscopy Analysis

After being polished with silicon carbide paper up to 4000 grit, the specimens were analyzed by confocal Raman microscopy to investigate the chemical composition of the reparative dentin bridge and primary dentin. Five specimens for every group and three analysis points were investigated for every area of interest.

The Raman spectra were collected using a Witec Confocal Raman Microscope System alpha 300R (Witec Inc., Ulm, Germany). The excitation for the confocal Raman microscope was provided by a frequency doubled Nd:YAG laser (Newport, Evry, France) at a wavelength of 532 nm, with 50 mW laser output power in a single longitudinal mode. The light was carried to the microscope by a single mode fiber (diameter of 25 µm). The incident laser beam was focused onto the sample through a 60× water immersion objective with a numerical aperture of 1.0 and a working distance of 2.8 mm (Nikon, Tokyo, Japan). The Raman backscattered radiation mixed with the Rayleigh scattered light were then passed through an edge filter to block the Rayleigh light. With the assumption that most of the Rayleigh light was filtered, the entire light was passed through to a multi-mode fiber (125 µm) directed to an Electron Multiplying Charge Coupled Device (EMCCD) camera (DU 970N-BV353, Andor, Hartford, CT, USA). The EMCCD chip size was 1600 × 200 pixels, the camera controller was a 16 bit A/D converter operating at 2.5 MHz, and the camera was cooled by a Pelletier system. The acquisition time of a single spectrum was set to 0.5 s. For experiments, 150 × 150 points per image were recorded using a piezoelectric table, leading to a total of 22,500 spectra for one image, with each spectrum corresponding to a spatial unit defined as a voxel. The data acquisition and processing were performed using Image Plus software from Witec (Ulm, Germany). The laser power on the sample was estimated to be less than 15 mW. 

## 3. Results 

### 3.1. Histological Evaluation of Reparative Dentin after Direct Pulp Capping

**Figure 1:**
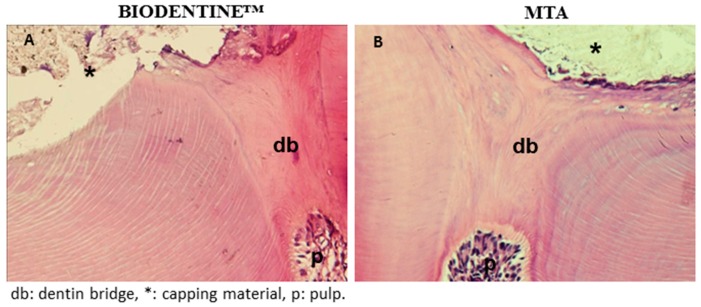
Hematoxylin eosin (HE)-stained sections of reparative dentin bridge: HE-stained sections of the Biodentine™ group (**A**) and the mineral trioxide aggregate (MTA) group (**B**) showed a homogeneous and continuous mineralized barrier between the dental pulp and the capping material.

The dentinal reparative bridge was shown to be complete when assessed after 4 weeks of implantation by HE staining. The reparative structures were homogenous, and in continuity with the primary dentin (Figure 1A,B). Adjacent pulp tissue appeared normal and free of inflammatory cells. This continuous reparative bridge was observed in MTA and in Biodentine™ capped groups. It is noteworthy that no tunnel defects were observed inside the new mineralized tissue

### 3.2. SEM of the Reparative Dentin

**Figure 2:**
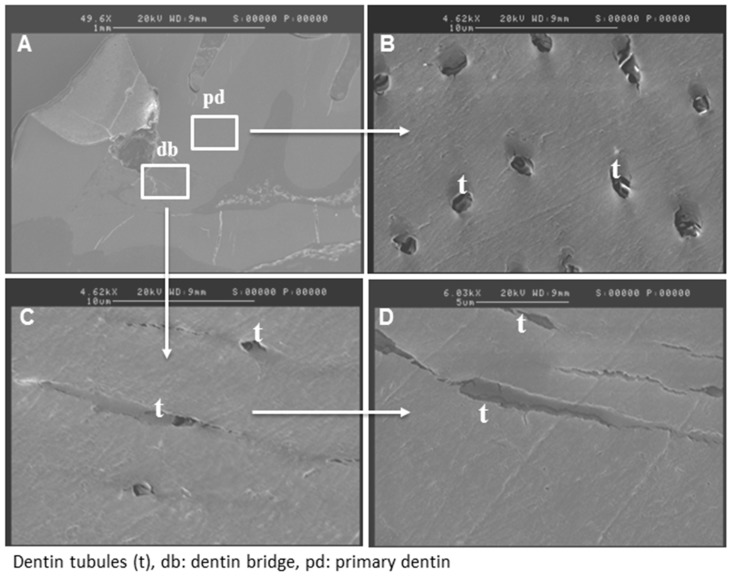
Scanning electron micrograph after direct pulp capping using Biodentine™. SEM analysis shows well-mineralized tissue near the pulp exposure (**A**) and at higher magnification, many dentinal tubules through the reparative dentin bridge can be observed (**C**,**D**). The number of dentinal tubules is lower than that of the primary dentin (**B**).

**Figure 3:**
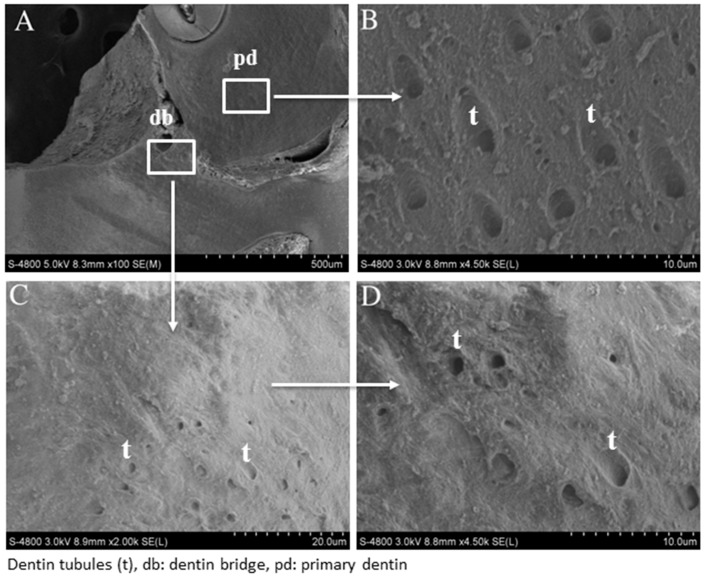
Scanning electron micrograph after direct pulp capping using MTA. SEM analysis shows a well-mineralized tissue near the pulp exposure (**A**) and at higher magnification, many dentinal tubules through the reparative dentin bridge can be observed (**C**,**D**). The number of dentinal tubules is lower than that of the primary dentin (**B**).

SEM analysis of reparative dentin bridge after MTA and Biodentine™ direct capping was performed. In the Biodentine™ group, a homogenous mineralized tissue was visualized next to the material (Figure 2A). At higher magnification, dentinal tubule structures were clearly observed through this new reparative tissue (Figure 2C,D). However, the number of tubules seemed lower than those in the primary dentin (Figure 2B), which served as a control. Similar results were obtained for the MTA group (Figure 3A–D).

### 3.3. EDX Microanalysis of the Reparative Dentin

X-ray microanalysis detected mineral charges in the dentin bridge for both groups. The mineralization level of the new reparative tissue is similar to the primary dentin level. Specifically, for the Biodentine™ group, the Ca /P ratio was measured at 1.75 in the dentin bridge and at 1.74 in the primary dentin. In the MTA group, the Ca /P ratio was measured at 1.69 in the dentin bridge and at 1.74 in the primary dentin (Table 1). 

### 3.4. Confocal Raman Analysis of Reparative Dentin

Chemical changes in samples could be traced by Raman spectroscopy. The Raman data analysis showed that the mineralized bridge of the MTA group, that of the Biodentine™ group, and the primary dentin have the same mineral phase and organic phase. 

**Figure 4:**
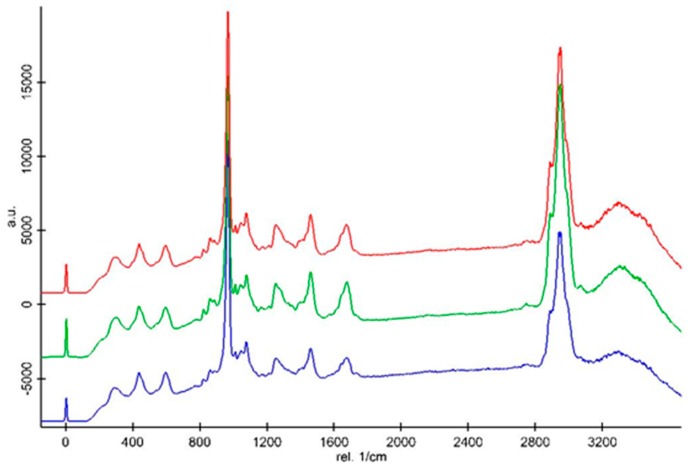
Representative Raman spectra of the dentin bridge of the MTA group (red line), the dentin bridge of the Biodentine™ group (blue line), and the primary dentin (green line).

Representative Raman spectra obtained from the dentin bridge of the MTA group (red line), the dentin bridge of the Biodentine™ group (blue line), and primary dentin (green line) are shown in Figure 4. These three structures have the same Raman peaks, which are related to the inorganic and organic matrices. The identification and assignment of the Raman peaks associated with organic and inorganic components of the secondary dentin are well documented [14]. The peaks associated with the inorganic mineral appear in the spectral range including the vibration of type B carbonate ν_1_ (CO_3_) at 1078 cm^−1^ and vibration peaks of phosphate groups—ν_1_ PO_4_^3−^ (first vibrational mode) at 960 cm^−1^; ν_2_ PO_4_^3−^ at 435 cm^−1^; and ν_4_ PO_4_^3−^ at 601 cm^−1^. The peaks associated with the organic component appear in the spectral range containing δ(CCH) aromatic (Pro, Tyr) at 854 cm^−^^1^, ν(CC) aromatic (Hyp, Tyr) at 875 cm^−^^1^, δ(NH) amide III at 1254 cm^−^^1^, δ(CH) deformation at 1450 cm^−^^1^, ν (C=O) stretch/amide I at 1670 cm^−^^1^, and ν(CH) symmetric at 2945 cm^−^^1^.

**Figure 5:**
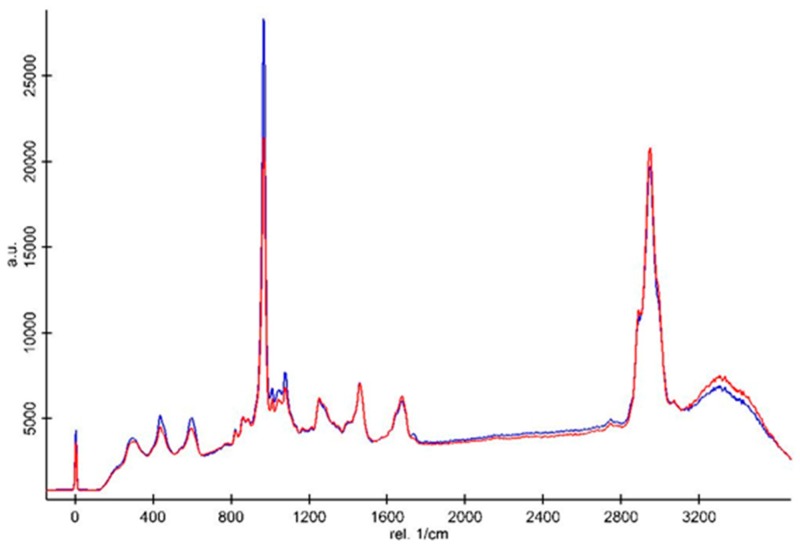
Representative Raman spectra of the dentin bridge (red line) and primary dentin (blue line) of the Biodentine™ group. The spectra were normalized based on the peak intensity of the CH_2_ bending vibration at 1450 cm^−^^1^.

**Figure 6:**
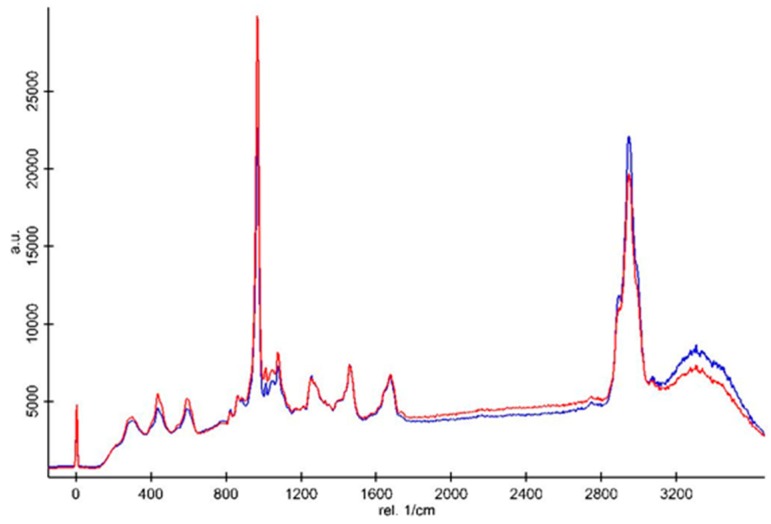
Representative Raman spectra of the dentin bridge (blue line) and primary dentin (red line) of the MTA group. The spectra were normalized based on the peak intensity of the CH_2_ bending vibration at 1450 cm^−^^1^.

All the spectra were normalized based on the intensity of the CH deformation peak at 1450 cm^−1^ (Figure 5 and Figure 6). Cosmic rays were removed using Witec software. The Raman spectra demonstrated that the intensities of the inorganic peaks including the vibration of type B carbonate and phosphate groups of the primary dentin were higher than those of the dentin bridge in both the Biodentine^TM^ (Figure 5) and MTA groups (Figure 6). The CH organic peak at 2945 cm^−1^ of the dentin bridge was slightly higher than those of the primary dentin in both material groups. However, only the intensity ratio of the phosphate peak at 961 cm^−1^ in the spectrum of the primary dentin was significantly stronger than those of the dentin bridge (Table 2).

## 4. Discussion

Direct pulp capping treatment is intended to preserve pulp vitality in selected cases. It has been shown that one of the most important properties of a pulp-protecting material is its capacity to induce the formation of high-quality mineralized tissue [15]. Though many studies have identified the tertiary dentin, most of them have only made a histological characterization this dentin. Some of them evaluated the volume of the formed mineralized dentin by cone beam computerized tomography [16,17]. None has really characterized the structural and chemical nature of the mineralized bridge. In this study, for the first time to the best of our knowledge, we evaluated the nature of the newly formed tissue after direct pulp capping using MTA and Biodentine™ by using electron microscopy and Raman spectroscopy. Based on the available data showing the good quality of the dentinal bridge obtained using these materials, our hypothesis in this current study was that the chemical nature of the reparative tissue is similar to that of normal dentin in our model. The reparative dentin bridge observed in both groups was similar to primary dentin.

The quality of a hard tissue bridge at the exposure site has been recognized as an important factor for the clinical success of direct pulp capping [18]. The main objective of an ideal capping material in exposed pulp might be complete reconstitution of the pulp periphery with a dentin-like matrix. In this study, after direct pulp capping using Biodentine™ or MTA, our first histological results confirmed the formation of a regularly dense mineralized tissue well-localized at the injured site. Interestingly, the thin necrotic layer between the pulp capping agent and the remaining vital pulp, seen in our previous study [3] at early time points, disappeared at 30 days. In fact, this layer—due to the released hydroxyl ions upon hydration during the calcium silicate setting reaction—was subsequently calcified by tertiary dentin formation from stimulated and differentiated dental pulp stem cells [19,20,21].

SEM findings confirmed a protective dentin bridge at the site of pulp exposure. At higher magnification, dentinal tubules were seen in the newly formed mineralized tissue, evoking a tissue resembling tubular dentin [22]. Electron probe microanalysis results revealed that the mineralization level of this new reparative tissue was similar to the level of primary dentin.

The approach used in the present study aims to further characterize the quality of the new mineralized tissue. Raman spectroscopy was used as a complementary method to SEM/EDX to draw a comparison between the chemical composition of the mineralized bridge of the MTA and Biodentine™ groups and primary dentin. In fact, confocal Raman microscopy could reveal a precise and reliable chemical comparison between samples. Raman spectroscopy is an analytical technique providing a high-resolution analysis [23]. Although the newly formed tissue is called the “reparative dentin”, its true nature was unclear. In our study, three structures including the dentin bridge of the MTA group, the dentin bridge of the Biodentine™ group, and the primary dentin had the same Raman peaks, which were related to the inorganic and organic matrices. In fact, these Raman peaks are similar to those of primary dentin [14]. The spectrum acquired for the primary dentin sample, with peaks at 602, 961, and 1078 cm^−1^, was similar to those recorded in previous studies reported in the literature [24], which assigned these peaks to the phosphate ion in hydroxyapatite. Similar peaks were also recorded for pure hydroxyapatite [25,26]. Peaks at 1254, 1450, and 1670 cm^−1^ were attributed to the organic collagen matrix of dentin [24]. However, slight changes of the first vibration of phosphate in the organic part in Biodentine™ and MTA were observed. This shows a higher ratio of phosphate in primary dentin compared to both material groups. 

Finally, our results showed for the first time that the chemical composition of the dentin bridge formed after direct pulp capping with MTA or Biodentine™ in rat models is similar to primary dentin. One bias of the present study is the pulpal status, which is not inflamed by a carious process. In fact, the reparative process could be different in the case of pulpal inflammation [27]. Some studies have shown that in teeth with carious pulpal exposures that were successfully capped with calcium hydroxide, the pulps were either uninflamed or infiltrated with scattered chronic inflammatory cells. The meticulous examination of serial sections revealed that all defects were repaired by the deposition of an amorphous, atubular calcified tissue, which could contain islands of entrapped necrotic tissues [27]. The point here was to investigate the use of calcium hydroxide as a pulp capping material. Our previous study [3] showed that CH is not a good material for inducing the formation of new mineralized tissue. Despite the inflammation level (which is a very good point) of this study, the use of CH is a bias as the low quality of the dentin bridge could be due to the material and not to the inflammatory status of the pulp. Another recent study showed that in humans with exposed pulps directly capped with Retro MTA, the new calcified hard tissue was not “regular dentin” and did not seem to be the product of genuine odontoblast differentiation [28]. These results suggest that the formation of calcified tissues after direct pulp capping with Retro MTA may be more appropriately regarded as a reparative process than as a genuine regeneration response. Interestingly, in our previous study, we showed that dentin tubules were observed and that the cells secreting the mineralized tissue displayed odontoblastic characteristics, such as a polarized shape and dentin sialoprotein (DSP) expression [3]. DSP is considered to be a specific biochemical marker of functional odontoblasts, with its role in dentin formation being an essential one, since it initiates and regulates biomineralization [29].

## 5. Conclusions

The results of this study suggest that the calcium-silicate-based cements used as pulp capping materials provide an optimal environment for pulp healing, resulting in a reparative dentin resembling on certain points primary dentin within the limitations of our rat model. 

## Figures and Tables

**Table 1:** Energy dispersive X-ray analyses of dentin bridge and primary dentin.

Material	MTA		Biodentine™	
Tissue	Dentin Bridge	Primary Dentin	Dentin Bridge	Primary Dentin
**Ca/P**	1.69 (0.06)	1.74 (0.06)	1.75 (0.08)	1.74 (0.07)

**Table 2:** The Raman intensity ratios of various peaks to the peak of CH_2_ at 1450 cm^−^^1^ from the dentin bridge and primary dentin.

Material	BIODENTINE™		MTA	
Various Ratios between Raman Peaks	Dentin Bridge Mean ± SD	Primary Dentin Mean ± SD	Dentin Bridge Mean ± SD	Primary Dentin Mean ± SD
R435/1454	0.83 (0.18)	1.16 (0.13)	0.69 (0.18)	1.01 (0.17)
R601/1454	0.68 (0.08)	0.89 (0.09)	0.51 (0.09)	0.75 (0.06)
R854/1454	0.58 (0.09)	0.53 (0.05)	0.5 (0.12)	0.48 (0.1)
R875/1454	0.12 (0.04)	0.14 (0.04)	0.1 (0.02)	0.11 (0.03)
R961/1454	9.26 (1.28)	11.2 (1.48)	7.02 (1.87)	10.26 (0.96)
R1078/1454	0.5 (0.13)	0.6 (0.15)	0.39 (0.09)	0.56 (0.08)
R1254/1454	0.93 (0.13	0.76 (0.04)	0.83 (0.14)	0.77 (0.11)
R1670/1454	0.88 (0.08)	0.68 (0.08)	0.89 (0.16)	0.76 (0.15)
R2945/1454	7.69 (0.64)	6.43 (0.74)	7.33 (0.93)	6.27 (0.39)
